# The Importance of Lactose in the Human Diet: Outcomes of a Mexican Consensus Meeting

**DOI:** 10.3390/nu11112737

**Published:** 2019-11-12

**Authors:** Enrique Romero-Velarde, Dagoberto Delgado-Franco, Mariana García-Gutiérrez, Carmen Gurrola-Díaz, Alfredo Larrosa-Haro, Ericka Montijo-Barrios, Frits A. J. Muskiet, Belinda Vargas-Guerrero, Jan Geurts

**Affiliations:** 1 Instituto de Nutrición Humana, Universidad de Guadalajara and Hospital Civil de Guadalajara “Dr. Juan I. Menchaca”, 44340 Guadalajara, Jalisco, Mexico; 2 Neonatology Department. ABC Medical Center, 01120 Mexico City and Instituto Tecnológico de Estudios Superiores de Monterrey, 64849 Monterrey, Mexico; ddelgadof@abchospital.com; 3 Endocrinology Department, Hospital Ángeles del Carmen. 44670 Guadalajara, Jalisco, Mexico; mariana.endoc@gmail.com; 4 Departamento de Biología Molecular y Genómica. Centro Universitario de Ciencias de la Salud, Universidad de Guadalajara, 44340 Guadalajara, Jalisco, Mexico; carmenhpv@yahoo.de (C.G.-D.); bevargro@hotmail.com (B.V.-G.); 5 Instituto de Nutrición Humana, Universidad de Guadalajara, 44340 Guadalajara, Jalisco, Mexico; alfredo.larrosa@academcos.udg.mx; 6 Servicio de Gastroenterología. Instituto Nacional de Pediatría, 04530 Mexico City, Mexico; erickamontijo@yahoo.com; 7 Laboratory Medicine, University Medical Center Groningen and University of Groningen, 9713 GZ Groningen, The Netherlands; f.a.j.muskiet@umcg.nl; 8 FrieslandCampina, 3818 LEAmersfoort, The Netherlands; jan.geurts@frieslandcampina.com

**Keywords:** lactose, lactose replacers, lactose intolerance, lactose maldigestion, lactase, galactose, microbiota, infants, children, adults

## Abstract

Lactose is a unique component of breast milk, many infant formulas and dairy products, and is widely used in pharmaceutical products. In spite of that, its role in human nutrition or lactose intolerance is generally not well-understood. For that reason, a 2-day-long lactose consensus meeting with health care professionals was organized in Mexico to come to a set of statements for which consensus could be gathered. Topics ranging from lactase expression to potential health benefits of lactose were introduced by experts, and that was followed by a discussion on concept statements. Interestingly, lactose does not seem to induce a neurological reward response when consumed. Although lactose digestion is optimal, it supplies galactose for liver glycogen synthesis. In infants, it cannot be ignored that lactose-derived galactose is needed for the synthesis of glycosylated macromolecules. At least beyond infancy, the low glycemic index of lactose might be metabolically beneficial. When lactase expression decreases, lactose maldigestion may lead to lactose intolerance symptoms. In infancy, the temporary replacing of lactose by other carbohydrates is only justified in case of severe intolerance symptoms. In those who show an (epi)genetic decrease or absence of lactase expression, a certain amount (for adults mostly up to 12 g per portion) of lactose can still be consumed. In these cases, lactose shows beneficial intestinal-microbiota-shaping effects. Avoiding lactose-containing products may imply a lower intake of other important nutrients, such as calcium and vitamin B_12_ from dairy products, as well as an increased intake of less beneficial carbohydrates.

## 1. Introduction

Lactose, as a unique carbohydrate in most mammalian milks, has been part of the human diet since our very origin. Nowadays, because of its chemical characteristics, lactose is found in many milk-derived products and is an important raw material in pharmaceutical products [1,2]. There are still questions, however, about the role of lactose (and thus dairy) in (1) infants and young children with symptoms of lactose intolerance, and (2) children and adults who are lactase non-persistent (LNP). LNP individuals hardly digest lactose and may show or develop symptoms of lactose intolerance [3,4,5].

Although dairy was introduced relatively late in the human diet, it appeared to be beneficial to its consumers, which resulted in initial mutations, leading to the ability to hydrolyze lactose lifelong (lactase persistence; LP). This created the opportunity to consume milk in relevant portions, thereby providing proteins of high biological value, and vitamins and minerals such as calcium [6,7]. Moreover, lactose in milk has a low glycemic index, which is generally considered a metabolic advantage. Recently, other benefits of lactose consumption via milk have been identified or suggested: shaping of the intestinal microbiota [8,9], exerting no neurologically rewarding effects when ingested [10,11], supporting immune function [12], and facilitating mineral absorption [13].

In recent years, the use of lactose-reduced or lactose-free infant formulae is, however, increasing in various countries, including Mexico. This is even the case for healthy infants and young children, who fully express the lactase enzyme and can, therefore, properly digest lactose. One of the contributing factors to this phenomenon could be the misdiagnosis of childhood functional gastrointestinal disorders (FGIDs) where complaints are incorrectly attributed to poor digestion of lactose. This, combined with the level of distress that the parents perceive in these situations, might explain, at least partly, why the transition to lactose-free or lactose-reduced formulae is so easily made [14,15]. Fear for "adverse" effects of milk/lactose consumption in early life may impact later stages of childhood and adolescence by reduction or elimination of milk from the diet, a habit that may lead to insufficient consumption of important nutrients needed for growth.

The purpose of the consensus meeting was to discuss and formulate a number of statements about lactose that represent the latest scientific insights and correct existing myths. This paper describes the discussions and provides the background on which the final statements were based.

## 2. Methods

To reach consensus on the nutritional and clinical importance of lactose, a total of 22 leading Mexican experts (representing the fields of pediatrics, gastroenterology, neonatology, clinical chemistry, genetics, endocrinology, and nutrition) from across the country, together with 2 experts from the Netherlands, convened in a 2-day meeting in Tequisquiapan, Querétaro, Mexico. Before the meeting and based on common questions about lactose, a set of statements was prepared. Next to that, 8 participants (topic leaders), considered to be experts in the field, were asked to prepare 30 min presentations on one of the statements. Presentations were given during the 2-day-meeting and each of the statements was extensively discussed within the group and amended where necessary. Consensus was achieved by incorporating all comments from participants into a written summary of the discussion for each topic. The summary documents were transformed into the present manuscript, including latest relevant publications, and were thoroughly reviewed by the topic leaders. The list of approved statements is presented in Section 3.14. Agreement on statements was given by each of the participants by email or oral communication.

## 3. Results

### 3.1. Lactose

The disaccharide lactose, with chemical structure galactose-β1,4-glucose, has been evolutionary conserved in virtually all placental mammalian milks, albeit in varying concentrations depending on the species [1]. Compared to milk from other mammals, human milk is considered unique in its high sugar content; it contains about 70 g/L lactose (7%) contributing around 40% of the caloric value [10]. In solution, it exists in two basic configurations, α and β-lactose, which are continuously converted into each other through a process called mutarotation. They differ in the position of the hydroxyl group on the C1 atom of the glucose molecule, but both configurations display identical digestion and metabolism characteristics in vivo [16]. The specific β-1,4 glycosidic bond between galactose and glucose is relatively rare in nature (although also present in cellulose and chitin). It may protect the lactating breast or neonatal gastrointestinal tract (GI tract) against infection by selective limitation of environmental microorganisms that have more difficulties with fermenting lactose compared to simple, alpha-linked glucose polymers. As such, lactose may be important in the early shaping of the neonatal gut microbiota [1]. The importance of lactose in the process of lactation is evidenced by various evolutionary adaptations by mother and child. In the mammary gland, lactose is completely synthesized from glucose provided by the arterial blood supply, and has low solubility (only 10% that of sucrose at 25 ˚C) but high stability in solution, thereby preventing lactose from diffusing out of the Golgi and secretory vesicles [17]. As a consequence, lactose can be easily secreted and aid in drawing water into these organelles, as such, determining the volume of the milk produced. The stability of lactose in solution enables the body to transfer significant amounts of energy to the neonate without excessive osmotic effects [17]. 

Several physiological benefits have been attributed to lactose in breast milk. Besides serving as an energy source, lacking any rewarding effects, unlike other carbohydrates such as sucrose or maltodextrin [10,11], it provides glucose and galactose, which serve as building blocks for various macromolecules (e.g., oligosaccharides, glycoproteins, and glycolipids) [18]. In addition, lactose is the least cariogenic amongst fermentable sugars [19], possesses a low glycemic index (GI = 46) [20] and can exert microbiota-modulating effects in the case of sufficient passage of undigested lactose into the lower intestine [8,9]. Furthermore, enzymatic hydrolysis into its monosaccharides is considered a re-hydration mechanism in the small intestine [21]. Lactose is widely used in food and pharmaceutical industries. Applications range from an energy source for lactic acid bacteria during dairy-product fermentation, in which its breakdown leads to the formation of specific flavor components, to its use as an excipient in oral, solid-dose pharmaceutical formulations [2].

### 3.2. Lactase (LCT)

Lactose needs to be broken down into its monosaccharides in order to be efficiently absorbed in the small intestine. Hydrolysis is controlled by lactase (LCT, also known as β-galactosidase or lactase phlorizin hydrolase (LPH)), a homodimeric enzyme anchored to the membranes of the small intestinal villus tips, with its highest expression being in the brush border of the mid-jejunum [22]. Maturation of the machinery for carbohydrate digestion and absorption occurs in a defined sequence during fetal development. Providing an adequate amount of nutrients is very important in order to reduce morbidity; however, the immature gastrointestinal system may represent a limitation. Therefore, understanding gastrointestinal development, which directly affects the metabolism of lactose, is key as it relates to the accretion of nutrients beneficial to the infant. Intestinal lactase activity is detectable in the fetal gut by eight weeks of gestation. Whereas sucrase, maltase, and isomaltase are usually almost fully active in preterm infants, lactase activity is low and proportional to gestational age (in infants <34 weeks, enzyme activity amounts to approximately 30% of that at term), but increases markedly from 24 to 40 weeks [23]. After the first feeding, there is a rapid increase in lactase activity to promote digestion of the lactose present in breast milk [24,25]; lactase-mediated hydrolysis of lactose typically exceeds 98% efficiency within five days of starting feeding [26]. Age, feeding, and nutrient composition have been shown to affect the development of intestinal lactase activity [27,28,29]. Premature babies that received early enteral feeding exhibited greater enzyme activity (100%) than infants in the control group (60%) [30]. Moreover, at 10 days old, lactase activity was higher in newborns that were fed with breast milk rather than formula [30]. Despite this relative “lactase deficiency” in the preterm infant, clinical symptoms of lactose intolerance are uncommon and the use of lactose is, therefore, not contraindicated [31]. Postnatal adaptive responses to carbohydrates ingested by the colonic microbiota further improve lactose digestion; even an excess of lactose that goes undigested by lactase in the brush border will be effectively fermented in the terminal ileum and colon by conversion into short-chain fatty acids [30]. During childhood and in a majority of children, lactase levels gradually decrease from a peak at birth to less than 10% of the preweaning level, with most of the change being observed at an age of about 2 years or older. However, lactase activity may persist into adulthood in some (sub)populations, which will be addressed in the next paragraph. Finally, congenital lactase deficiency has been described, but is rare; therefore, nutritional restriction of lactose is generally not necessary in term infants and children [4,28].

### 3.3. Development of Lactase Persistence (LP)

Physiological down-regulation of lactase expression by 90%–95% occurs in the majority of humans (~65%) post-weaning in the absence of intestinal injury or disease, a wild-type condition known as lactase non-persistence (LNP) [3,32]. Lactase activity persists, however, in some populations, a condition known as lactase persistence (LP), which can be attributed to (differential) regulation of lactase biosynthesis. Historically, the practice of pastoralism with its associated increase in consumption of milk and milk-derived products triggered this development of LP. These are all relatively recent events which significantly impacted our culture, biology, genetics, and behavior. LP is the most strongly selected single-gene trait currently known that evolved in Europeans and some African and Asian groups over the last 10,000 years [33,34,35,36,37]. The estimated spread of LP in Europe is thought to have been a very fast process. In fact, it has been calculated that the percentage of the population surviving to reproductive age increased by 5%–10% each generation due to the development of this trait, indicating that LP must have provided strong health benefits [6,7]. To date, it is unclear when LP exactly developed; data suggests anywhere between 4000 and 7500 years ago, probably in Central Europe [6]. Early evidence of animal domestication in southwestern Asia extends back to around 12,000 years ago. While initially, domestic animals were exclusively exploited for their primary products in the early Neolithic era, the widespread use of their secondary products—particularly milk—followed immediately or very soon after [38,39,40]. Dairying seems to predate the development of LP by several millennia, which might be explained by the initial consumption of fermented milk products, as these contain reduced lactose levels, causing no intolerance symptoms. Thus, in the developing pastoral societies of Eurasia, LP may have evolved as an adaptation to the consumption of fresh dairy products, thereby improving daily nutrition. Considering the varied ecologies in which LP evolved, it is unlikely that one single hypothesis explains the strong signatures of natural selection inferred across multiple geographical regions.

### 3.4. Regulation of Lactase Expression

Lactase is composed of two monomers, each consisting of two catalytic sites: domain III (phlorizin-hydrolase; beta-glucosidase activity) and domain IV (lactase; beta-galactosidase activity) [41]. LCT is located on the long arm of chromosome 2, is composed of 17 exons [3], and is primarily regulated at the transcriptional level [32]. The current view is that after lactase activity has been fully established, it is not influenced by dietary changes and/or lactose intake any longer [32]. Rather, differential lactase expression (as previously indicated, highest in the mid-jejunum) is orchestrated by gut-region-specific expression of the many transcription factors [22]. On a molecular level, epigenetic silencing in the condition of LNP takes place in both an upstream enhancer, located in intron 13 of the “DNA replication licensing factor MCM6” gene [42], and the promoter region of LCT itself [43,44]. LP is caused by well described single nucleotide polymorphisms (SNPs) in the enhancer, and these genetic changes prevent the epigenetic silencing of lactase after weaning [42]. Interestingly, in some populations, substrates for the phlorizin hydrolase domain of LCT might have driven development and evolutionary selection of LP. In these LP populations, a variety of triggers for lactase biosynthesis might have provided individuals the capability to detoxify certain nutrients (e.g., phlorizin) or increase the bioavailability of beneficial substrates like flavonoids (e.g., quercetin) [45]. Although the prevailing view is that there are no lactase-inducing or silencing signals from the environment, accumulating evidence (epigenetics and in vitro studies [46,47,48]) suggest that this cannot be excluded. 

### 3.5. Lactose Maldigestion and Intolerance, and Lactose Replacements

The non-immune mediated intolerance to carbohydrates is primarily due to maldigestion because of deficiency of enzymes or transporters, or is caused by overloading of intestinal brush border digestive/transport systems. Non-digested/-absorbed carbohydrates are readily fermented by the intestinal microbiota, resulting in the production of fermentation products, such as lactate, short-chain fatty acids (SCFAs), and several gasses (CO_2_, H_2_, and CH_4_). Severe maldigestion of carbohydrates may cause osmotic diarrhea [5,49,50]. 

Symptomatic lactose intolerance is the result of an impaired lactase capacity to properly digest lactose [3]. Congenital lactase deficiency, is an extremely rare, autosomal recessive condition caused by mutations in the lactase gene resulting in complete lactase absence [51,52]. Early in life, it is hypothesized (but unproven) that lactose maldigestion may, in rare cases, be caused by temporary lactase insufficiency (developmental or neonatal lactase deficiency) which generally resolves at three months of age, coinciding with the period of colicky behavior, although the role of lactose in infantile colic is controversial. Secondary lactase deficiency is a temporary decrease in lactase activity due to, for instance, damage of the intestinal villi of the small intestine. This may occur at any age, both in LNP and LP individuals. In the majority of people, and most often from late childhood onwards, lactose malabsorption has a genetic background (primary, adult-onset lactase deficiency) [4,5]. Lactose maldigestion and/or lactose malabsorption may result in clinical symptoms, such as distension of the small bowel, non-focal abdominal pain associated with bloating and flatulence, nausea, increased gut motility, and diarrhea. Additional systemic symptoms, such a headache, vertigo, memory impairment, and lethargy, have been described in less than 20% of subjects. It has been hypothesized that these symptoms could be caused by toxic metabolites derived from sugar and amino acid fermentation [50]. It is noteworthy that, out of these two types of fermentation, carbohydrate fermentation is linked predominantly to metabolic benefits of the host, whereas the more distal colonic fermentation of peptides and proteins is more closely associated with the production of harmful metabolites [53].

Although lactose maldigestion has been proposed as a factor involved in functional gastrointestinal disorders (FGIDs) [54,55,56,57,58,59], no conclusive evidence for a direct relation has been shown [4,26,60,61]. In order to address lactose intolerance (maldigestion) and associated (clinical and nutritional) issues, lactose replacement and restriction strategies have widely received attention. According to the CODEX Alimentarius [62], glucose polymers can replace lactose in infant formulae to meet the caloric requirements. Based on effects of glucose and sucrose overconsumption in children, it has been hypothesized that regular consumption of lactose substitutes in infant formulae may represent a potential risk factor for the development of overweight or obesity. The following paragraphs will address the various aspects involved in the diagnosis, functional consequences, and treatment of lactose intolerance. In addition, the short and long-term effects of lactose replacements on key physiological parameters will be highlighted.

### 3.6. Diagnosis of Lactose Intolerance

The incidence of lactose intolerance depends on the diagnostic method used and the amount of lactose used in the test. While a lactase activity assay in jejunal biopsies is the gold standard for diagnosis, the lactose hydrogen (H_2_) and/or methane (CH_4_) breath test is more practical and less invasive [63,64,65]. To discriminate between hydrogen and methane producers, and fast or slow fermenters, a pre-screening test with lactulose (always fermented) can be performed [66]. Finally, lactose exclusion or reduction (during 2–4 weeks) to achieve symptomatic improvement, followed by gradual lactose reintroduction, has proven useful and is often applied [66]. With regard to the dose of lactose used for intolerance tests, the commonly-used dose of 50 g may not be realistic, since this is the equivalent of 1 liter of milk, which is highly unlikely to be consumed at once in practice. Therefore, 25 g might represent a more suitable provocation dose, at least for adults [67]. An even better alternative might be the implementation of a dairy product tolerance test (e.g., drinking 250 mL of milk) with symptom evaluation up to 24 hours post-consumption and the inclusion of an H_2_/CH_4_ breath test [68,69]. For infants and children, a H_2_/CH_4_ breath test and elimination-provocation diets, using physiological doses (2 g/kg of body weight) of lactose, are commonly used [4,60].

### 3.7. Possible Consequences of Lactose Restriction

Once lactose intolerance has been diagnosed, it is important to identify its severity and underlying cause. Most infants digest lactose very well and a lactose-restricted diet is only necessary for a limited period of time in case of transient lactase deficiency (due to, for instance, intestinal epithelial damage). In subjects with LNP (typically older children and adults), varied amounts of lactose are still tolerated without the presentation of significant symptoms. Generally, individuals with LNP can consume up to 12 g of lactose in a single dose with no or only minor intolerance symptoms [5,70]. In addition, the oral administration of β-galactosidase (exogenous lactase) can improve tolerance to lactose-containing foods [5]. In the absence of formal guidelines, the American Academy of Pediatrics and the National Institutes of Health published general recommendations for the management of lactose intolerance [60,71]. 

Currently, no data exist on the long-term effects of lactose restriction in children. However, since lactose is a unique and major carbohydrate in mammalian milk, the exclusion of lactose containing milk and dairy-derived products also implies a restriction of the other nutrients present in these products. Dairy products are important sources of bioavailable calcium, vitamin D (when added), and vitamin B_12_, and, over time, lactose-restriction may induce insufficiency, potentially resulting in decreased bone mineral density, anemia, and neuropathy [5,72]. Overall, depending on the severity of intolerance symptoms, a temporary reduction in lactose can be beneficial; however, this is no reason to stop breastfeeding. As previously indicated, lactose maldigestion due to LNP develops in a major part (65%–70%) of the global population from (late) childhood onwards and can, but certainly does not always, lead to symptoms of lactose intolerance. 

### 3.8. Lactose Maldigestion and Functional Gastrointestinal Disorders (FGIDs)

Lactose maldigestion develops when the amount of ingested lactose exceeds the capacity of lactase activity. As indicated above, most infants and toddlers can properly digest lactose and the likelihood of lactose maldigestion without the presence of intestinal damage factors, such as infection or allergy, is very low. In addition, there is no robust evidence to suggest that infants and toddlers with FGIDs present lactose maldigestion [4,26,60,61]. Under specific circumstances, however, gasses produced during lactose fermentation can cause or worsen abdominal pain and distension [4,60]. In patients with irritable bowel syndrome and visceral hypersensitivity, a reduction of lactose and/or other carbohydrates of the FODMAP (fermentable oligosaccharides disaccharides monosaccharides and polyols) group has shown a significant reduction in abdominal pain and bloating [54,55,56]. It should be noted that the current explanation for the occurrence of FGIDs in children and adolescents is not that of a single-cause entity; FGIDs are recognized and identified by morphological and physiological abnormalities that often occur in combination with other morbidities, including motility disturbances, visceral hypersensitivity, altered immune function, altered microbiota composition and activity, and altered central nervous system processing [57]. Additionally, stool reducing substances, i.e., unabsorbed reducing sugars in the stool, are not associated with the occurrence of colic. In fact, they are frequently present in the stools of healthy infants, either fed with human milk or lactose-containing formula, under 10 weeks of age. The presence of reducing substances in the stool of infants fed with human milk is even four times higher than that of formula-fed infants, probably due to the presence of a substantial amount of human milk oligosaccharides [26,61,73]. In formulae, prebiotics may explain part of the reducing sugars, but the presence of some lactose cannot be excluded. Nevertheless, reducing sugars do not appear to be harmful or associated with FGIDs. Taken together, lactose should not be considered a hazardous nutrient [74].

### 3.9. Lactose Replacements: Metabolic Consequences of Glucose Polymers 

In lactose-free or lactose-reduced infant formula, lactose is substituted by glucose polymers. The most common glucose polymers are digestible maltodextrins, glucose syrups, and dried glucose syrups; these glucose polymers are produced by the hydrolysis of different types of starches (corn, rice, or potato starch) [75,76]. Starch, maltodextrins, glucose, and lactose all have a similar energy value (4 kcal/g), but differ in the rates of digestion and absorption. Glucose is immediately available for absorption, whereas starches, maltodextrins, and lactose need to first be digested by α-amylase, maltase, and lactase, respectively [76]. Of note, the enzymatic digestion of maltodextrins occurs at a high rate, resulting in post-ingestion insulin responses comparable to pure glucose. 

Glucose polymers have a higher glycemic index (GI = 110) than lactose (GI = 46), and as a result, induce a higher glycemic response. The low GI of lactose is primarily caused by the non-insulinogenic response to galactose. This could be a metabolic advantage compared to glucose polymers that cause a larger increase in plasma glucose and plasma insulin. For adults, this appears to be true; GI is a valid parameter to classify diets with regard to their preventative (low GI) or provoking (high GI) effect on diabetes, coronary heart disease, and likely obesity as well [77]. For children and adolescents, such (preventive) low GI benefits are still under discussion. A study in infants on the effects of consumption of lactose or corn syrup-containing formula did not reveal any differences in insulin or blood glucose levels (based on area under the curve) over a two-hour postprandial period. However, after two hours, the lactose group exhibited significantly higher glucose and insulin levels compared to breastfed and corn syrup-containing formula groups [78]. It is possible that the increased glucose levels (120 min postprandial) that were observed were partially due to both glycogenolysis (breakdown of (galactose-derived) glycogen) and gluconeogenesis [79,80]. Another study, using urinary C-peptide to creatine ratio as a stable marker of insulin secretion, showed a higher C-peptide secretion after the consumption of a corn-sugar based formula compared to a lactose based formula, indicating higher insulin levels in the former [81]. 

High blood glucose levels are involved in protein glycation, the non-enzymatic reaction between glucose (or other reducing sugars) and an amine group from free amino acids or proteins. Although initially, glycation of molecules is reversible, irriversible complexes, called advanced glycation endproducts (AGEs), are formed over time. AGEs accumulate with age and are linked to certain complications associated with diabetes, kidney disease, metabolic disorders, and degenerative diseases [82,83]. Regular consumption of considerable amounts of high GI carbohydrates, that substantially increase blood glucose levels, enhances the risk of AGE formation [84]. 

Limited evidence [85] suggests that maltodextrin, consisting of α-1,4 and α-1,6-linked D-glucose chains, may disrupt host-microbial dynamics in the gut when it escapes digestion, and may be associated with the promotion of *Escherichia coli* colonization. Furthermore, it was shown that in vitro cellular exposure to maltodextrin leads to impaired anti-bacterial responses, as demonstrated by the increased viability of intracellular *Salmonella* in macrophages and epithelial cells cultured in maltodextrin-supplemented media. Although the effect of maltodextrin on *Salmonella* clearance in macrophages was dose-dependent, any exposure was sufficient to increase bacterial viability [85]. In another animal study, it was recently shown that maltodextrin promotes endoplasmic reticulum stress in intestinal Goblet cells, which leads to mucus depletion and exacerbation of intestinal inflammation [86]. Therefore, when providing a lactose-free formula that contains maltodextrins, changes in microbiota should be carefully monitored to get more insight in this topic. It is of interest to note that the gut microbiota is increasingly recognized as an import part of our bodies and that changes, and the intestinal microbiota are linked to various physiological processes, including body weight management [87]. An increased Firmicutes/Bacteriodetes ratio and a reduced capacity to degrade carbohydrates to short-chain fatty acids is related to metabolic dysfunction of the host organism [88]. This also highlights the importance of maximizing efforts (via, e.g., low heat treatment) to limit glycation of proteins during the production process of products containing proteins and reducing sugars (such as lactose). These food-derived glycated proteins may contribute to overall obesity risk, as the majority are not digested but fermented [83].

With regard to minerals, lactose is known to enhance the absorption and retention of calcium, magnesium, and manganese; this is related to a pH drop due to its fermentation in the large intestine, which increases solubility of calcium (and other minerals), thereby increasing passive absorption. However, the functional relevance of this increased absorption is questionable, considering the surplus of calcium that is typically added to formulae designed for infants and young children [13].

Ultimately, the risk of detrimental long-term effects on health due to providing high GI carbohydrates early in life should be carefully considered; replacement of lactose with glucose polymers might lead to a worse nutritional status, a change in intestinal microbiota, and reduced calcium absorption. The effects of lactose-containing or lactose-free formula on insulin and glucose levels in infants is less clear and largely inconclusive. Evidence for short-term effects on parameters linked to chronic diseases is very limited. However, since nutritional preferences are shaped during childhood, the long-term relevance of early exposure to sweet, high GI food products may be considerable [77,82].

### 3.10. Lactose Replacements: Taste Preference and Obesity Risk Later in Life

Behavioral studies have indicated that in the last trimester of pregnancy, the taste buds are able to detect and send information to the central nervous system regarding the composition of the amniotic fluid, which illustrates that the development of the taste sense occurs at a very early stage in life. Similarly, it has been shown that at birth, children have a preference for sweet flavors, including carbohydrates in breast milk, which allows them to identify the sources of energy necessary for growth [89]. This preference for sweet flavors persists during childhood and adolescence and can potentially be a disadvantage in view of the obesogenic environment in which we live. Indeed, the availability and consumption of a large number of industrialized products, including sugary drinks, has been associated with the development of overweight and obesity in children and adolescents [90]. Interestingly, the sweetening effect of lactose is weak and devoid of any neurochemical stimulation (e.g., lactose does not evoke rewarding effects) [91]. The lack of a neurochemical response may explain why feelings of pain in infant rats are suppressed by glucose, sucrose, and fructose, but not by lactose [92]. The pain-reducing properties of sucrose have been replicated in human newborns [93]. 

The use of infant formulae as substitutes for breast milk is increasingly common, particularly in countries like Mexico, where parts of the population have abandoned the practice of breastfeeding and the recommendations of the WHO [94]. Infant formulae are often subject to several modifications, such as the hydrolysis of proteins, or the partial or total replacement of lactose with other carbohydrates, such as maltodextrins, glucose syrups, and even sucrose [95]. Like many other countries in the world, Mexico suffers from an epidemic of overweight and obesity amongst children and adolescents (prevalence of around 35%). Although the taste for sweet flavors is innate (at least to some extent), it has also been pointed out that regular consumption of sweetened foods and drinks can have adverse short and long-term consequences on food preference and lead to the consumption of excessive amounts of sugar [96]. Unfortunately, a lot of uncertainty exists regarding the regular consumption of formulae with lactose substitutes due to a shortage of literature on this topic. Thus, at this time, it cannot be affirmed that regular consumption of infant formulae containing lactose replacers is a risk factor for the development of overweight or obesity. Studies comparing regular consumption of these formulae to consumption of other foods are warranted. Eventually, assessing if the accumulation of adipose tissue differs between children who consume infant formulae with and without lactose would be of great value. It should be noted that, besides the potential differential effects of lactose and lactose substitutes on food preference, lactose possesses other properties that can contribute to healthy body weight development. Lactose has a low GI, a property that has been shown to play a subtle role in the prevention or treatment of childhood obesity [77]. Furthermore, if undigested lactose reaches the lower intestine, the fermentation of lactose generates short-chain fatty acids that have been shown to regulate body weight through effects on food intake and energy metabolism [97]. Recently, partial replacement of glucose with galactose, resulting in a 1:1 ratio mimicking lactose, in a 3-week post weaning diet, was shown to lower body weight, adiposity, homeostatic model assessment of insulin resistance (HOMA-IR), and expression of insulin signaling in high fat diet-challenged female mice in later life. These findings suggest that prolonged galactose intake may improve metabolic and overall health for extended periods of time through galactose-mediated nutritional programming [98]. 

### 3.11. Potential Health Benefits of Lactose as a Supplier of Galactose and via Microbiota Shaping Effects

The gastrointestinal tract is colonized by different microorganisms, which participate in the mucosal immune system through diverse mechanisms [99]. Although the GI tract microbiota’s composition, origin and abundances are controversial, evidence suggests that important microbiota-shaping factors include the maternal microbiota (oral, intestinal, and vaginal), circumstances and inoculation at birth (hospital, home, and normal versus caesarean delivery), anti-biotic treatment, and food (milk) composition. In addition, the period preceding delivery may be important, since bacteria seem to be present in the placenta, amniotic fluid, and meconium [100,101,102]. Gut microbiota and the host have a symbiotic relationship, with the former strengthening the immune system and protecting the host against pathogens [102]. Qualitative and quantitative changes in the intestinal microbiota, its metabolic activity, and its local distribution, affect pathobionts and pathogens in the gut, a state known as “dysbiosis.” These changes could contribute to the development of diseases, such as colorectal cancer, gastrointestinal disorders, obesity, allergy, and others [102,103,104,105,106]. Recently, the genus *Bifidobacterium* has gained growing interest as it has been associated with the presence of undigested lactose and several health benefits [104]. Other factors, such as stress, diet, and genetic background, may also impact *Bifidobacterium* prevalence in the gut [107]. In the next paragraphs, we will discuss our current knowledge on the (potential beneficial) effects of (undigested) lactose on *Bifidobacteria* and the microbiota in total. In addition, the specific relevance of galactose downstream of lactose in glycogen synthesis and possibly as an important building block for glycosylation will be addressed as well.

### 3.12. Lactose, Bifidobacteria, and the Microbiota: Potential Health Benefits?

Breast milk is essential in infancy because it supplies important biomolecules, including oligosaccharides, antioxidants, IgA, and anti-inflammatory compounds, that offer protection against diseases [105]. As indicated previously, lactose is normally cleaved into the monosaccharides glucose and galactose by lactase in the GI tract [67]. However, if undigested, lactose reaches the terminal ileum and colon (as explained earlier), and it is fermented by the intestinal microbiota, favoring colonization by *Bifidobacteria* and other lactic acid bacteria. Additionally, in vitro studies have revealed that lactose increases the levels of gastrointestinal antimicrobial peptides. The colonization of *Bifidobacteria* and the induction of antimicrobial peptides both potentially protect, albeit independently, the neonatal gut against infection [31]. In LNP subjects (lactase <10 u/g) and in infants that are physiological malabsorbers of lactose, lactose can exert microbiota-modulating effects as well [8,9,108]. 

Lactose is fermented by members of the microbiota that can easily split the β-1,4-glycosidic linkage. Studies in weaning pigs show a positive, dose-related effect of dietary supplementation with lactose on intestinal *Bifidobacteria* and *Lactobacilli*, and an increase in total volatile fatty acids in feces with a particular increase in butyric acid [109]. In infants, the addition of lactose (38 g/L) to an extensively hydrolyzed formula resulted in significantly augmented counts of *Bifidobacteria* and lactic acid bacteria, and decreased numbers of *Bacteroides* and *Clostridia* compared to a lactose-free variant. The same study also demonstrated a positive effect of added lactose on the fecal metabolome with increased concentrations of short-chain fatty acids (mainly acetic and butyric acid) [110]. In young adults with lactose malabsorption (as indicated by increased exhaled hydrogen levels), whole milk supplementation (250 mL/day, during 4 weeks) resulted in a higher relative abundance of fecal *Actinobacteria*, *Bifidobacteria*, *Anaerostipes* (butyrate producers), and *Blautia* (acetogenic) compared to normal lactose digesters [111]. Next to lactose, human milk oligosaccharides (HMO) are well-known stimulators of the neonatal intestinal microbiota [112]. Additionally, prebiotics in infant formulae show microbiota-shaping effects (i.e., an increase in *Bifidobacteria* up to levels seen in breastfed infants, accompanied by a smaller rise in *Lactobacilli*) [113,114]. Interestingly, galacto-oligosaccharide (GOS) supplementation appears to be beneficial for lactose-intolerant individuals [115,116]. Stimulation of both *Bifidobacterium* and *Faecalibacterium* activities ameliorated lactose-intolerance symptoms [117]. In fact, both a *Bifidobacterium*-rich fecal microbiota, associated with reduced lactose digestion, together with increased levels of short-chain fatty acids, exerted a protective effect on colonic mucosal integrity and early immune system development [118]. Less well-known fibers, such as fructans from Mexican Agave plants, may exhibit these microbiota-shaping effects as well and could support the proper fermentation of lactose [119]. 

As discussed earlier, lactose may also have immunomodulatory effects. Postpartum, infants do not have fully functional adaptive immune systems and rely on the innate immune system with its antimicrobial peptides and proteins expressed on epithelial surfaces. In vitro research suggests that lactose may be part of this early defense system. Lactose (supplied as the hydrophilic fraction of breast milk or as commercially-available purified lactose) induced, in vitro, the *cathelicidin antimicrobial peptide* (*CAMP*) gene encoding the human antimicrobial peptide cathelicidin LL-37 in colonic epithelial cells in a dose and time-dependent manner. Lactose also induced *CAMP* in the colonic epithelial cell line T84, THP-1 monocytes, and macrophages. Furthermore, a synergistic effect on CAMP induction was demonstrated with butyrate and phenylbutyrate. Although infant formulae also showed a stimulation of *CAMP*, a dose-dependent effect was lacking [31]. These antimicrobial peptides, such as CAMP, might play a role in further shaping the neonatal microbiota [12]. 

Many of the proposed beneficial effects of lactose can be linked to short-chain fatty acids that (1) serve locally as energy for the gut microbiota and the cells of the intestinal wall, (2) confer a protective effect on colonic mucosal integrity, (3) exert a beneficial effect on early immune development, and (4) are used as energy source following absorption and transport to the liver [31,120]. Overall, it appears that lactose can beneficially influence the gut microbiota and play a role in the development of *Bifidobacteria* in the guts of LNP individuals, infants, and children. Interestingly, *Bifidobacteria* metabolize lactose to short chain fatty acids and lactate, but not H_2_, whereas H_2_ can comprise up to 50% of the flatus volume [121]. Therefore, its consumption/supplementation, albeit in adequately sub-threshold doses with respect to lactose intolerance complaints, can lead to improved lactose tolerance. 

### 3.13. Potential Health Benefits of Galactose

It seems unclear why nature designated lactose as the disaccharide to uniquely occur in mammalian milk; its synthesis requires substantial investment of energy by the mother, and the breastfed infant subsequently requires energy for its digestion. Part of the explanation could be that coupling of the two monosaccharides glucose and galactose, reduces the osmotic pressure of milk, which by definition is equal to the osmotic pressure of maternal plasma. Coupling allows for a higher mother-to-child transfer of a carbohydrates that serves as a slow-release energy source and a provider of building blocks for complex macromolecules in the infant. Following lactase-mediated hydrolysis of lactose in the infant’s GI tract, both glucose and galactose are predominantly taken up by the sodium-glucose linked transporter-1 (SGLT1) [122,123]. This symporter also transports sodium and water, and co-transports calcium by bulk flow [124]. Unabsorbed lactose, as well as unabsorbed glucose and galactose moieties and HMOs may serve as substrates for the microbiota in the infant’s colon [125]. Most of the absorbed glucose passes the liver to elicit an insulin response, which causes the majority of galactose to become trapped in the liver [126], where it becomes a major substrate for the synthesis of glycogen [18,123,126]. The glucose-mediated insulin-response enhances glycogen synthesis by inhibiting a kinase or kinases, such as glycogen synthase kinase-3 (GSK-3) [127], that inactivate glycogen synthase [123,126,128]. Although galactose is not considered an essential nutrient in general, it could be conditionally essential in infants in the context of rapid growth. Galactose is used for the synthesis of a variety of oligosaccharides, glycoproteins, and glycolipids [129]. Moreover, lactose-derived galactose could be important in assuring metabolic flexibility in infants/young children, because it behaves in a non-insulinogenic manner (under fed conditions), and thereby lifts the break (e.g. insulin) on fat metabolism [78]. This enables the infant to switch to fat metabolism (e.g., ketone body production) when there is a high demand for energy, for instance during rapid growth. Amongst others, ketone bodies are a great energy substrate for the brain, which is incapable of oxidizing fats itself.

Thus, lactose limits milk osmotic pressure, promotes uptake of sodium, water and calcium, and acts as a microbiota-modulating nutrient, partially as unabsorbed lactose, and partially as a source of building blocks for the more complex HMOs. Absorbed glucose and galactose serve as the foundation for macromolecules, a rapidly available energy source (glucose), and ‘storage’ in the form of mobilizable glycogen in the liver (generated from galactose) [18]. 

### 3.14. Consensus Statements

The participants of the consensus meeting adopted the following statements:

Lactose does not differ in biochemical-structural composition between mammalian milks, and it is a unique carbohydrate in nature with beneficial properties in most people.

Lactase activity is detectable in the fetal gut by eight weeks of gestation; and at least from 32 weeks of gestational age onwards until late childhood or longer, lactose (6–7 g/100 mL) can be digested sufficiently.

Lactase expression in case of primary, adult-onset lactase deficiency, does not stop at a precise moment in time: its down-regulation is the result of an incompletely understood, internal, epigenetic developmental program. 

Lactase persistency is a very strongly and positively selected genetic trait that significantly increased survival rates after it arose, amongst others, by improving daily nutrition. Nowadays, approximately 35% of the world’s population is lactase persistent.

Lactose maldigestion (any undigested lactose) is not a clinically relevant issue in infants and children, unless symptoms of lactose intolerance or symptoms related to GI diseases, such as celiac or Crohn’s disease, are severe.

Lactose maldigestion is not a harmful process when the dose does not exceed the fermentation capacity of the intestine and is not related to functional gastrointestinal disorders.

Lactase non-persistent children and adults still can consume lactose without symptoms of intolerance but the tolerable amounts are variable and depend on several factors. It is said that up to 12 grams of lactose in a single dose is well tolerated, and when combined with a meal and/or distributed over the day, even higher amounts are well tolerated by many maldigesters.

Lactose contributes to the development of a *Bifidobacteria*-rich microbiota and may act as a microbiota-shaping carbohydrate especially in LNP persons but also in LP infants and children.

Dietary galactose, in combination with glucose (simultaneously delivered to the body, in the case of lactose), plays an important role in infant’s and children’s nutrition in the replenishment of liver glycogen.

Exogeneous galactose supply (e.g., via lactose) may be needed under conditions of growth and development (e.g., infants and young children) as a structural building block for glycosylated macromolecules (e.g., galactocerebrosides for myelination by oligodendrocytes in the brain).

The consumption of high GI maltodextrins and the consecutive rise in blood glucose levels may not be favorable for infants and children in the long run because of its impact on inflammatory pathways.

Lactose is preferred over lactose replacers in infant’s and children’s nutrition because of its weak sweet taste and the fact that lactose consumption does not elicit any rewarding effects. Lactose could, therefore, lead to less imprinting of sweet taste at a young age, and therefore, reduce the preference for sweet flavors later in life. 

The potentially beneficial impact of lactose on overweight development in children is at the moment, not scientifically validated. Specifically-designed longitudinal scientific studies are needed to clarify this potential link.

## 4. Discussion

Lactose, synthesized in the mammary gland, is nature’s way to get a significant quantity of carbohydrates in milk, and largely determines the volume of milk produced and provides a relevant portion of slow-release energy [17]. Since lactose is the primary carbohydrate in most mammalian milks and its concentration in milk cannot be influenced by the maternal diet, it is obvious that lactose is the preferred carbohydrate in infant and young child formulae. However, the use of lactose-reduced or lactose-free formulae in several countries is increasing. The main driver for this is the often-false coupling of symptoms of intestinal discomfort to (perceived) lactose intolerance. From childhood onwards, lactose maldigestion is established in the majority of the world’s population because of a genetically-driven down-regulation of lactase expression (LNP) [3,32]. The proposed benefits of lactose (energy source without rewarding effects, supplier of galactose and glucose, low GI, microbiota-modulating activity, and low carcinogenicity) [8,9,10,11,20,21] necessitate a careful, well thought-out approach for managing lactose maldigestion. During this workshop, the attendees discussed the available evidence for the proposed benefits of lactose, the clinical aspects of lactose intolerance, and the importance of lactose for infants, children, and adults, both in the context of LNP and LP. This resulted in the drafting of the 13 statements presented in this document (see Section 3.14. Consensus Statements).

When lactose is digested, the absorbed glucose passes the liver to elicit an insulin response, which causes the majority of galactose to become trapped in the liver [126], acting as a major substrate for the synthesis of liver glycogen [18,123,126]. Any toxic effects of galactose, as supplied by lactose, are neglectable. Even after the ingestion of 100 g of lactose, the maximum concentration of galactose in the systemic circulation stays ≤1 mmoL/L [79]. Taking into account that the maximal recommended daily dose of galactose is 50 g for a healthy adult, liver galactose clearance capacity appears to be high enough under normal circumstances [130]. However, the ingestion of galactose without glucose or with alcohol consumption induces higher, non-pathological plasma concentrations of galactose [79]. In the liver, galactose is converted by the enzyme galactokinase into galactose-1-phosphate, which is converted into uridine diphosphate (UDP)-galactose, UDP-glucose, and next into glycogen, or it is transformed into glucose-1-phosphate [79,80,131]. Pathologically elevated blood galactose levels may occur because of deficient functioning of enzymes involved in galactose metabolism (e.g., GALK (galactokinase), GALT (galactose-1-phosphate uridyl transferase), or UDP-Galactose-4 epimerase (GALE)), a rare condition known as galactosemia [132]. Individuals suffering from this disease accumulate certain galactose metabolites (galactitol and galactose-1-phosphate) leading to damage of the eye lens, liver, kidneys, and brain. As a source of glucose, liver glycogen can restore postprandial blood glucose levels when these become too low. Although galactose is also used for the synthesis of a variety of oligosaccharides, glycoproteins, and glycolipids [129], it is not known to what extent dietary galactose is used for this. At least in adults, the use of dietary galactose seems to be limited and most galactose is produced endogenously from glucose. This is not, per se, the case in infants and young children since they have much higher growth-related demands. Lactose intake has been indicated to favor brain function in children; however, it is not clear if this can be directly linked to the systemic galactose availability [133]. Lactose-derived galactose could be important in assuring the metabolic flexibility required in infants/young children, because it acts in a non-insulinogenic way (under fed conditions), and thereby lifts the ban (i.e., insulin) on fat metabolism [78]. Additionally, for adults, the non-insulinogenic characteristic of lactose-derived galactose can be of interest in light of metabolic health. 

An often-mentioned benefit of lactose is its low glycemic index (GI). There is convincing evidence from meta-analyses of controlled dietary trials that diets low in GI improve glycemic control in people with type 2 and type 1 diabetes. There is also convincing evidence from meta-analysis of prospective cohort studies that low GI/GL diets reduce the risk of developing type 2 diabetes [77]. Consumption of lactose-containing dairy products is neutral or moderately beneficial to type 2 diabetes risk [134]. Dairy consumption is sometimes linked to an increased type 1 diabetes risk, especially in young children at risk (genetic predisposition for type 1 diabetes). Mechanistically, this is linked to dairy proteins, specifically to A1 β-casein and its seven amino acid peptide β-casomorphin-7 (BCM-7) derivative, but not to lactose. The ecological relationship of milk A1 β-casein with type 1 diabetes across populations is strong. However, a causal role in its own right is unlikely [135]. There is a similar relationship with vitamin D status, for which the putative mechanistic background in (auto)immune disease is plausible [136,137]. Given the association of low vitamin D status with many other immunologically-driven diseases, it seems possible that in the etiology of type 1 diabetes mellitus, a low vitamin D phenotype interacts with A1 β-casein/BCM-7 or some other antigen(s), to which the gut mucosa is exposed in early life. Interference with mucosal tolerance might be key. Recently, an international, randomized clinical trial in 2159 infants at risk to present the disease, randomly assigned to the consumption of a formula based on extensively hydrolyzed casein versus a formula with intact milk protein, found no difference between groups in the incidence of type 1 diabetes after a median follow-up of 11.5 years [138].

Lactose maldigestion is a result of too much lactose relative to lactase capacity. Lactase expression reaches mature levels around term age [27,28,29]; this means that newborns and young children can digest physiological amounts of lactose (i.e., as present in breastmilk) without any problems. Only when they have no or low lactase expression due to congenital lactase deficiency, gastrointestinal infections, or to preterm birth, can the digestion of lactose be compromised. Although LNP children and adults have a considerably limited capacity to digest lactose, most of them should be able to deal with the fermentation of 12 g of lactose in a single dose without having any relevant symptoms of lactose intolerance [5,70]. In about 35% of the world’s population, lactose can be digested, as lactase expression is continuous (LP) due to SNPs that prevent the epigenetic silencing of lactase after weaning. No conclusive evidence is available for a direct relationship between lactose intolerance and FGIDs [4,26,60,61]. Exclusion of lactose containing milk and derived dairy products also implies a restriction in the intake of other nutrients present in these products.

Widely-used glucose polymers in lactose-free infant formulae are digestible maltodextrins and glucose syrups. Additionally, sucrose is sometimes used. It is unclear if these lactose replacements are nutritionally equivalent to lactose, except regarding calories. When comparing lactose to sucrose, various differences can be observed: (1) lactose contains glucose and galactose, while sucrose contains glucose and fructose. Galactose and fructose have different metabolic behaviour inside the body; (2) lactose contains a β1,4-glycosidic linkage, while sucrose contains an α1,2-glycosidic bond; (3) the GI of lactose is 46, while the GI of sucrose is 65; (4) lactose has low sweetness and lacks rewarding effects after consumption, while sucrose possesses high sweetness and elicits rewarding effects; (5) lactose contains the versatile monosaccharide galactose which sucrose lacks; (6) lactose is less cariogenic than sucrose; and (7) lactose exerts gut-microbiota-modulating effects that lead to increased uptake of calcium, an effect not found for sucrose. Limited evidence suggests that maltodextrin is also not equivalent to lactose. Maltodextrin, consisting of α-1,4 and α-1,6-linked D-glucose chains, may disrupt host-microbial dynamics in the gut when it escapes digestion, and may be associated with the promotion of *E. coli* colonization. A decision to replace lactose should take these differences into account. 

## 5. Conclusions

Lactose is the preferred carbohydrate for infants and young children, for reasons that it (1) acts as an excellent, slow release, energy source; (2) possibly contributes (in infants) to the synthesis of complex glycosylated macromolecules by the provision of the building blocks glucose and galactose; (3) elicits no reward effects after consumption; (4) has a low cariogenic effect (not discussed in this paper); and (5) exerts gut microbiota-shaping effects, most likely promoting a more saccharolytic than proteolytic microbiota. Though some degree of lactose maldigestion will take place in all infants and young children, this will seldomly lead to lactose intolerance symptoms. Additionally, in in LNP adults and older children, dose-dependent fermentation of lactose takes place without symptoms of intolerance almost always, while providing all the benefits of this fermentation. Considering all above-mentioned benefits, the replacement of lactose by other carbohydrates has to be considered with care and moderation.
